# Endoscopic Diagnosis and Treatment of Superficial Esophageal Squamous Cell Cancer: Present Status and Future Perspectives

**DOI:** 10.3390/curroncol29020048

**Published:** 2022-01-26

**Authors:** Ryu Ishihara

**Affiliations:** Department of Gastrointestinal Oncology, Osaka International Cancer Institute, Osaka 541-8567, Japan; ryu1486@gmail.com; Tel.: +81-6-(6945)-1181; Fax: +81-6-(6945)-1900

**Keywords:** esophageal cancer, cancer invasion depth, endoscopic resection, curability assessment, ESD

## Abstract

This review provides information regarding the preoperative examinations, indications for endoscopic resection (ER), and curability assessment in subjects with superficial esophageal squamous cell carcinoma (SCC). Narrow-band imaging (NBI) is a more sensitive modality for detecting esophageal cancer than conventional observation, and esophageal observation using NBI is thus recommended for the detection of superficial esophageal cancer. It is also important to adjust the volume of air in the esophagus during observation. Workup by non-magnifying followed by magnifying endoscopy is a common process for diagnosing the invasion depth of superficial esophageal SCCs in Japan. Endoscopic ultrasonography carries a risk of overdiagnosis, and its routine use is therefore not recommended. The Japanese endoscopic submucosal dissection/endoscopic mucosal resection guidelines for esophageal cancer considered the indications for ER based on the results of studies focusing on clinical MM/SM1 cancers, and concluded that clinical MM/SM1 carcinomas, except circumferential carcinoma, were an indication for ER. The curative effect of ER should be assessed based on histologic examination of the resected specimens. ER should be conducted based on a thorough understanding of the preoperative diagnosis, indication, curability, and additional treatment of esophageal SCC.

## 1. Introduction

Esophageal cancer is the seventh most prevalent cancer and the sixth most common cause of cancer-related mortality worldwide [1]. While esophageal squamous cell cancer (SCC) is the most prevalent histological subtype worldwide, dominant in Asia and Africa, there is an increasing prevalence of adenocarcinoma in western countries where this subtype is more prevalent than squamous cell cancer [1]. More than half of esophageal SCC in Japan are detected as advanced cancers, and only about 37% are detected as superficial cancers [2].

Advanced esophageal SCC has a poor prognosis, but cancers detected at a superficial stage may have a favorable prognosis if treated with endoscopic treatment, chemoradiation, and surgical resection [3,4,5,6]. Endoscopic treatment is the preferred treatment option due to its minimally invasive nature. In this manuscript, we review the current status and future prospects of endoscopic treatment for superficial esophageal SCC.

## 2. Detection and Diagnosis of Esophageal SCC

### 2.1. Modalities Used for Detection

Early stage esophageal SCC can be recognized by a slight redness, loss of mucosal luster, and areas of low vascular permeability using white-light imaging. However, many esophageal SCCs are difficult to detect using white-light endoscopy [7], and various efforts have been made to overcome this difficulty. Narrow-band imaging (NBI) [8], developed in the early 2000 s, irradiates narrow-band light to highlight the mucosal surface, making it possible to detect esophageal SCCs that are difficult to detect using white-light endoscopy [7].

NBI is more sensitive for detecting esophageal SCC than white-light endoscopy, and is thus recommended for the detection of superficial esophageal SCC. In addition, it is important to adjust the volume of air in the esophagus during observation, given that esophageal lesions are more visible when the esophageal wall is less-extended (less-air) compared with when the esophageal wall is fully extended (normal-air) [9].

### 2.2. Endoscopic Diagnosis of SCC

**Figure 1:**
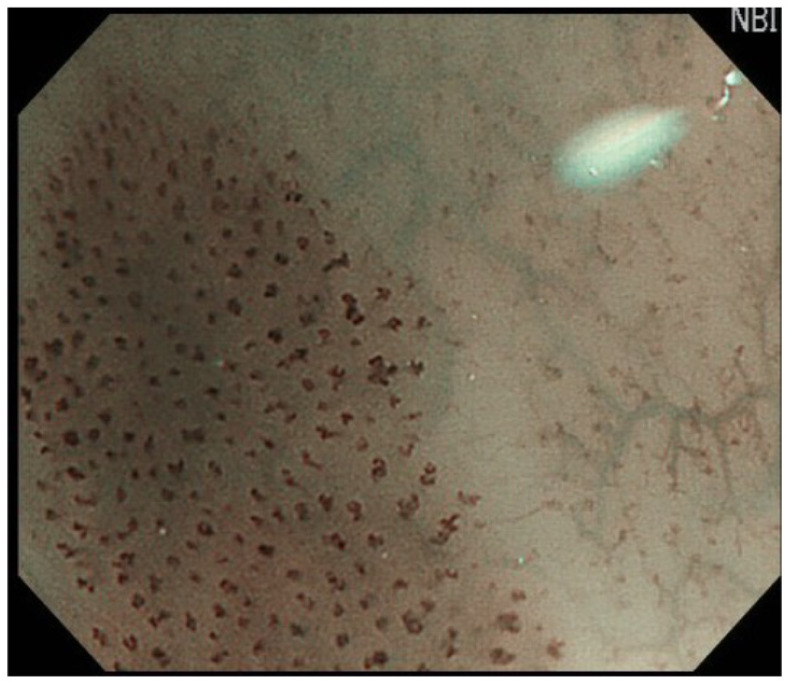
Dot-like changes in blood vessels are seen in addition to background coloration.

Superficial esophageal SCC often appears as a brownish area on NBI. Brownish areas in the esophagus should thus first be observed closely, and dot-like changes in blood vessels, in addition to background coloration, indicate a high likelihood of esophageal SCC (Figure 1) [10]. Magnifying endoscopy allows a more-detailed evaluation of the blood vessels, and a diagnosis of esophageal SCC is usually made based on the Japanese Esophageal Society (JES) classification [11]. Type B1 vessels are dilated, tortuous vessels of the intra-epithelial papillary capillary loop with non-uniform caliber and shape, and are characteristic of superficial esophageal SCC, especially if they are limited to the epithelium (EP) or invade into the lamina propria (LPM). In contrast, the presence of vessels lacking all four of these characteristics (Type A vessels) often indicates a non-cancerous lesion, such as esophagitis or intraepithelial neoplasia.

**Figure 2:**
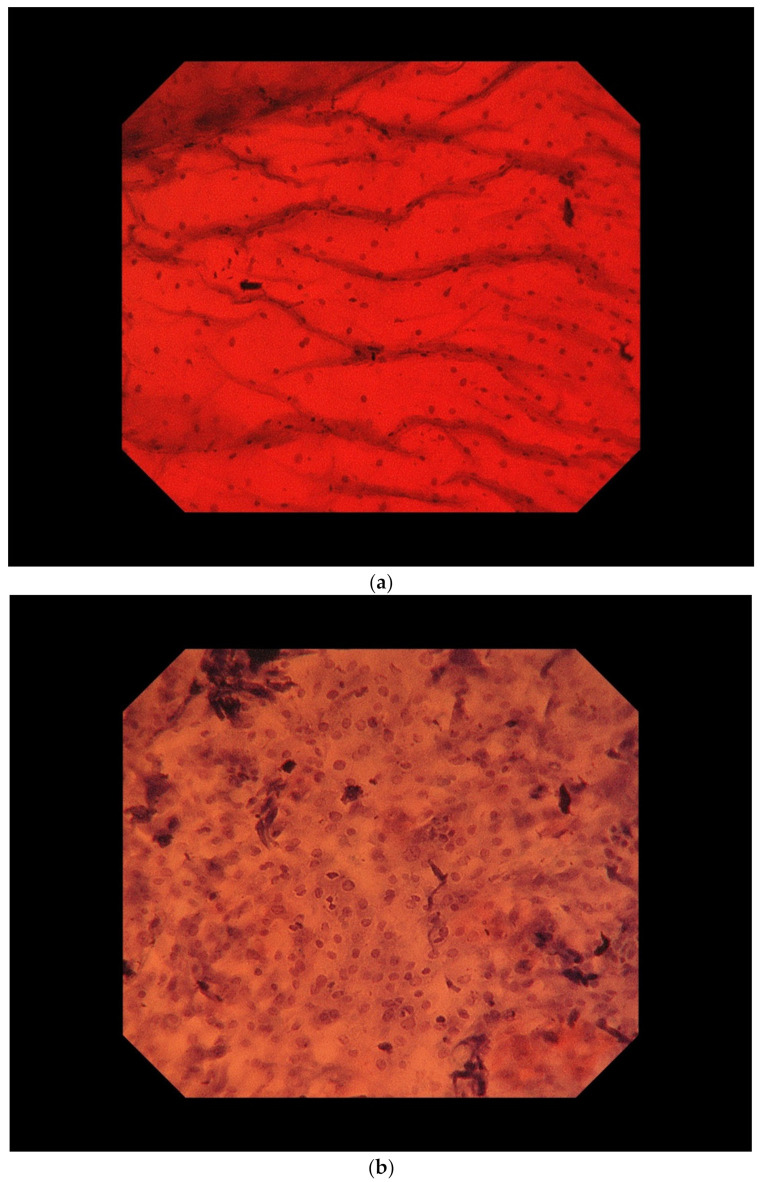
Ultra-magnifying endoscopic images of non-cancerous mucosa (**a**) and esophageal cancer (**b**).

Magnifying endoscopes with a magnification function of about 100× have been used since around 2000 to observe microvessels, such as in the intra-epithelial papillary capillary loop. However, an ultra-magnifying endoscope with an optical magnification function of up to 520× was introduced into routine clinical practice in 2018, allowing the evaluation of cellular atypia in the esophageal mucosa following staining with methylene blue or toluidine blue. In the normal esophageal mucosa, surface differentiation occurs towards the mucosal surface, with the cells becoming thinner and the nuclei becoming more concentrated. In esophageal SCC however, the esophageal surface is often replaced by cancer cells, and cancer cells with a high nuclear/cytoplasm ratio and nuclear atypia can be observed by ultra-magnifying endoscope (Figure 2). This endoscope thus allows the real-time evaluation of nuclear atypia in vivo.

## 3. Endoscopic Diagnosis of Cancer Invasion Depth

### 3.1. Modalities Used for Diagnosis of Cancer Invasion Depth

Superficial esophageal cancer depth can be divided into EP/LPM, muscularis mucosa (MM)/submucosa (SM)1 (invading submucosa by ≤ 200 µm), and SM2 (cancer invading into the submucosa > 200 µm). Cancers diagnosed as EP/LPM and MM/SM1 are often treated by endoscopic resection (ER), while cancers diagnosed as SM2 or deeper are often treated with surgical resection or chemoradiation therapy [12,13]. It is therefore important to differentiate between SM1 and shallow cancers, and SM2 and deeper cancers to ensure appropriate treatment selection.

Conventional white-light endoscopy, magnifying endoscopy, and endoscopic ultrasonography (EUS) have been the main techniques used for diagnosing cancer invasion depth [14]. However, a recent study reported no significant improvement in diagnostic accuracy when additional EUS was performed after conventional white-light endoscopy and magnifying endoscopy, while overdiagnosis, suggesting a deeper than actual cancer depth, was increased [15]. Overdiagnosis may lead to more invasive treatments such as esophagectomy and chemoradiotherapy for cancers that could be cured by ER (overtreatment). Considering EUS increases the incidence of overdiagnosis, it is therefore preferable to diagnose the depth of superficial esophageal cancers by conventional white-light endoscopy and magnifying endoscopy.

### 3.2. Diagnosis of Cancer Invasion Depth by White-Light Endoscopy and Magnifying Endoscopy

Cancer invasion depth is initially observed using non-magnifying white-light endoscopy to evaluate findings such as surface irregularity and thickness. Cancers with an almost flat or slightly uneven surface (≤1 mm) are considered to be SM1 or less, while cancers with an elevation >1 mm or a lesion thickness >1 mm are diagnosed as SM2. When magnifying endoscopy is used, the invasion depth is diagnosed based on the JES classification. If the entire lesion is composed of Type B1 vessels or avascular area (AVA)-small, the cancer is diagnosed as EP/LPM; if Type B2 vessels or AVA-middle are observed, the cancer is diagnosed as MM/SM1; and if Type B3 vessels or AVA-large are observed, the cancer is diagnosed as SM2.

## 4. Indications for ER 

### 4.1. Cancer Invasion Depth

ER is indicated in patients with T1N0M0 esophageal squamous cell carcinoma based on the invasion depth and lateral extent of the cancer (Table 1). EP/LPM cancers are considered suitable for ER due to their very low metastatic risk, while MM/SM1 carcinomas are regarded as a relative indication for ER, considering the risk of metastasis of pathological (p)MM/SM1 cancers [12]. However, because the indication for ER is based on preoperative diagnosis, the endoscopic submucosal dissection (ESD)/endoscopic mucosal resection (EMR) guidelines for esophageal cancer [13] investigated the indications for ER based on the results of studies focusing on clinical (c)MM/SM1 cancers. Previous studies showed that 27.4–55.2% of patients with a preoperative diagnosis of cMM/SM1 had pEP/LPM cancers that have low risk of metastasis, and if ER produced non-curative results, a favorable outcome could be anticipated with appropriate subsequent therapy. cMM/SM1 carcinomas, except for circumferential carcinoma, are thus regarded as an indication for ER in the ESD/EMR guidelines for esophageal cancer [13].

### 4.2. Lateral Spread of SCC

ER is a good treatment, esophageal ER for large lesion can lead to post-ER esophageal stricture, with proportion of 60.7–75% for non-circumferential but >3/4 and 100% for whole-circumferential resection without any preventions [16,17,18]. However, effective preventions can decrease the proportion of strictures to 11.3–36.2% [16,17,19]. Non-circumferential lesions are thus regarded as an indication for ER. Considering that >70% of cEP/LPM cancers with circumferential extent are pEP/LPM [20] and the risk of stricture is relatively low in lesions < 50 mm, the guidelines recommend ER with stricture prevention for cEP/LPM < 50 mm in length and circumferential lesions [13] (Table 1).

### 4.3. ER Procedure

**Figure 3:**
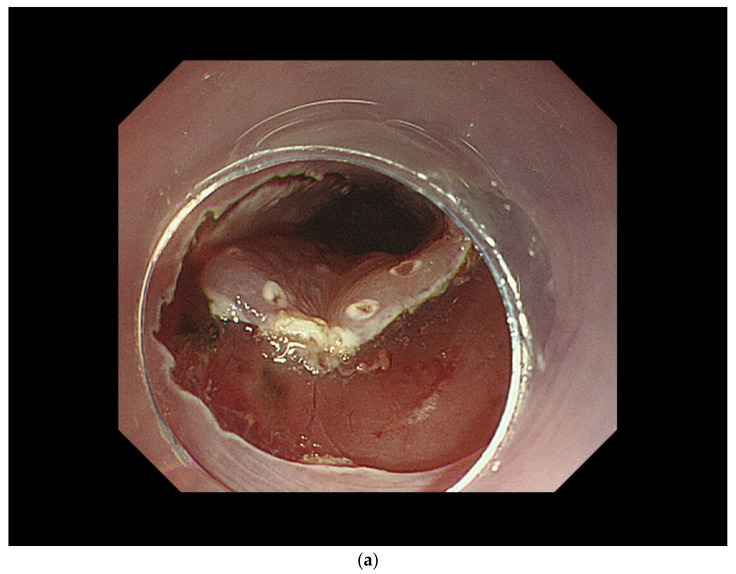

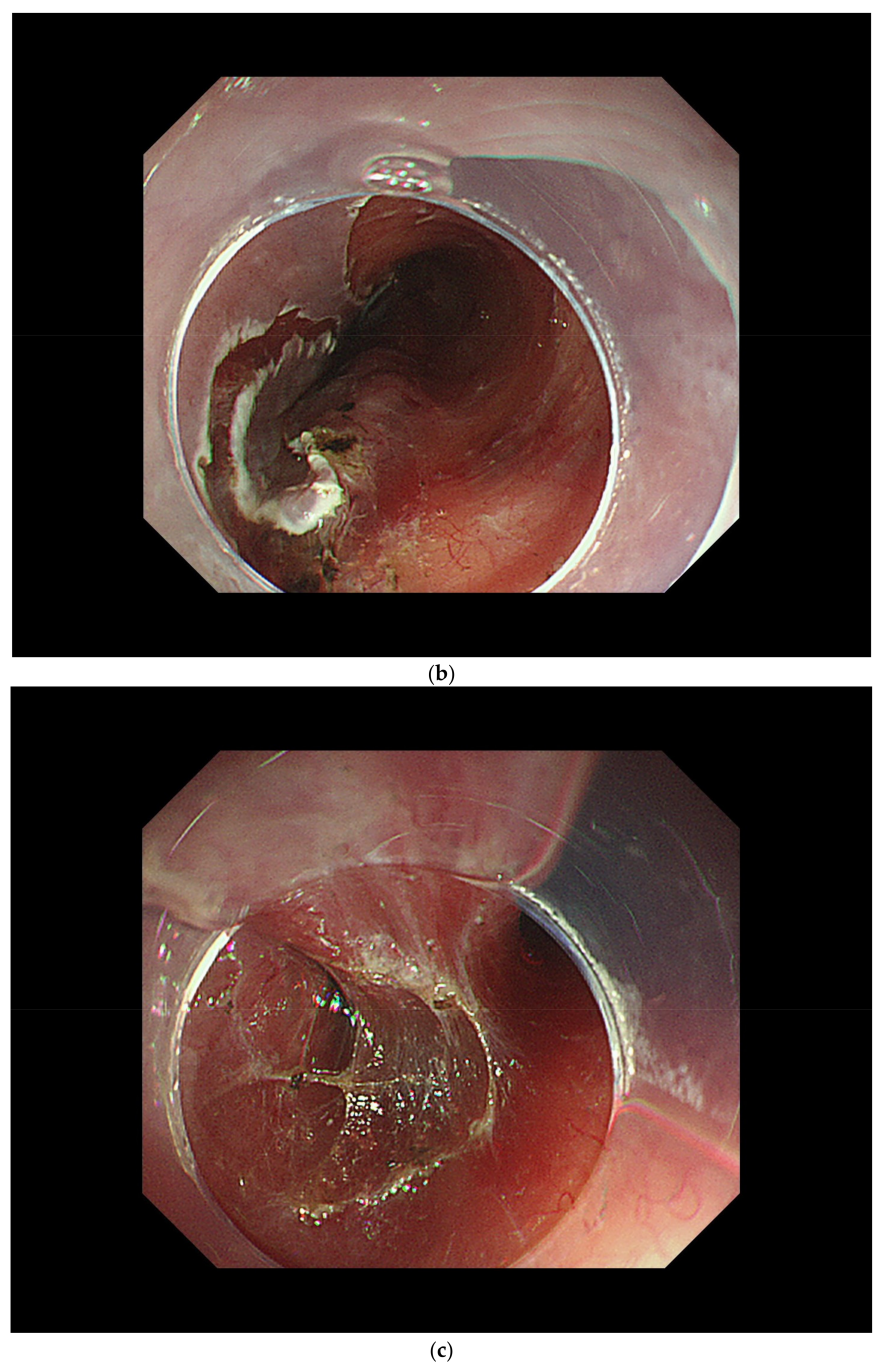

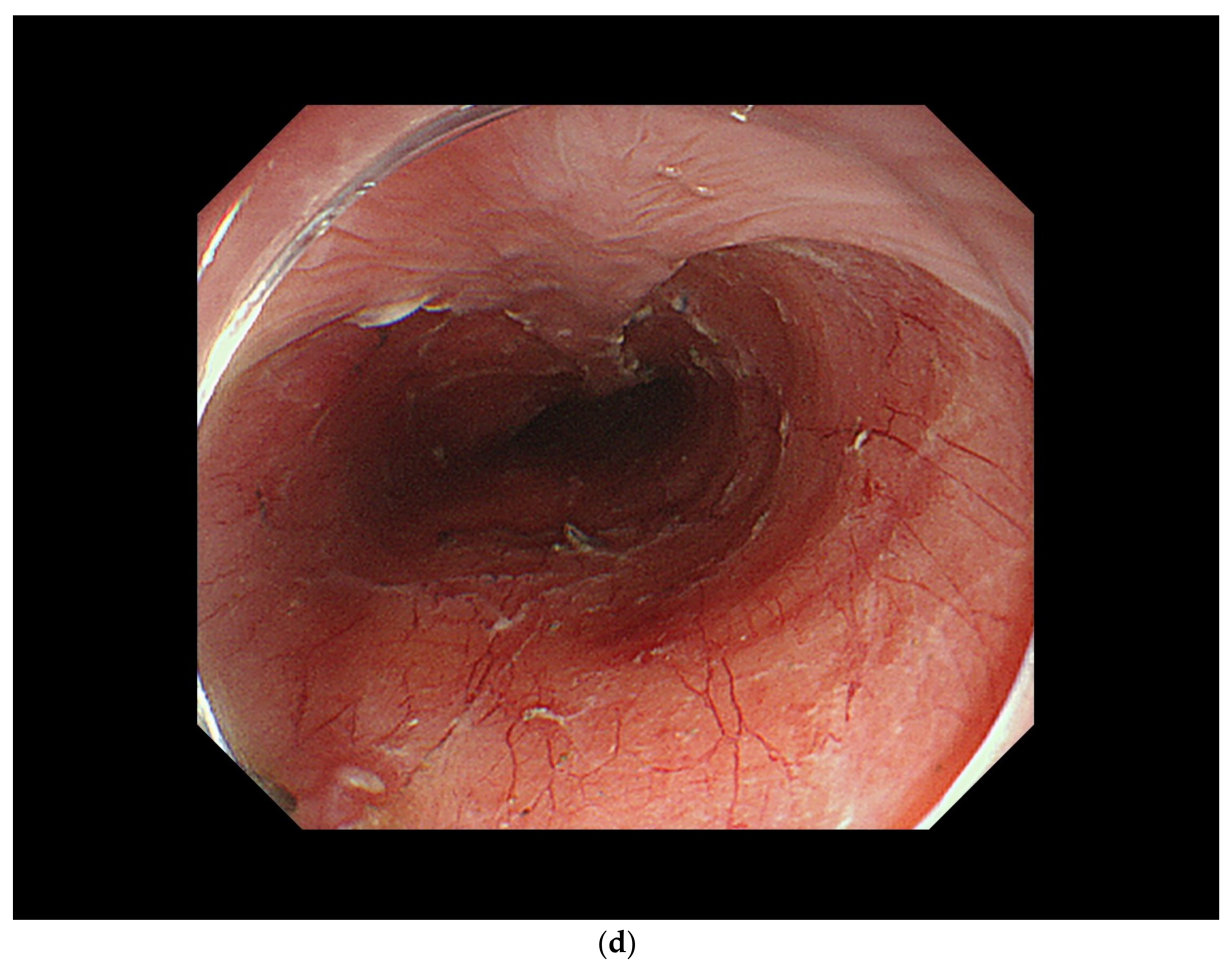
Process of endoscopic submucosal dissection. (**a**) Mucosal incision of the lesion. (**b**) Submucosal dissection of the lesion (right side). (**c**) Submucosal dissection of the lesion (left side). (**d**) Total removal of the lesion.

ER of esophageal SCC can be performed by either EMR or ESD, with the latter becoming the main approach in recent years. In ESD, the mucosa surrounding the lesion is incised and the submucosa beneath the lesion is dissected to remove the lesion, thus enabling en bloc resection of the lesion. There are two representative ESD procedures: the tunnel method and the C-shaped method (Figure 3). A clip with a thread is attached to the mouth end of the lesion and the thread is gently pulled out of the mouth to provide stable traction on the submucosa. This method is useful for shortening the procedure and improving safety. When >75% of the esophagus has been resected, local injection or oral administration of steroids is used to prevent esophageal stricture.

## 5. Curability Assessment 

### 5.1. pT1a-EP/LPM Cancer without Lymphovascular Invasion

The metastatic rate of pT1a-EP/LPM cancer without lymphovascular invasion is very low [4], and resection is thus considered as curative (Table 2).

### 5.2. pT1a mm Cancer without Lymphovascular Invasion

The guidelines [13] tabulated the metastatic frequencies of pT1a mm and lymphovascular invasion-negative cancers, which indicated a metastatic rate for pT1a mm cancer without lymphovascular invasion of 5.6% (12/216 patients) in the absence of additional treatment after ER. Considering the relatively high risk of metastasis, pMM cancer without lymphovascular invasion was considered non-curative (Table 2). However, considering the risk of additional treatments, these are not conducted in general.

### 5.3. pSM Cancer or pMM with Lymphovascular Invasion

The proportions of metastasis in subjects with pMM and lymphovascular invasion have been reported to be as high as 21.4% (3/14 cases) and 25.3% (43/170 cases) in pSM1 cancer, and 25% (49/196 cases) in pSM2 cancer. Given the relatively high risk of metastasis, pSM cancers or cancers with positive lymphovascular invasion are considered non-curative, and additional treatments such as esophagectomy or chemoradiation are required (Table 2).

## 6. Comparison of ER and Surgical Resection

There are two papers comparing the outcomes of ESD and esophagectomy for pT1 esophageal SCC. A report from China [21] found no meaningful difference between the groups in mortality, mortality by esophageal SCC, or proportion of metastasis, even after adjusting relevant factors. However, there was a lower number of subjects with treatment-related mortality in the ESD group compared with the esophagectomy group, without statistical significance (0.3% vs. 1.5%; *p* < 0.186). In addition, high-grade adverse events in the ESD group was significantly lower than the esophagectomy group (15.2% vs. 27.7%; *p* < 0.001).

In another report [22], mortality, mortality by esophageal SCC, or survival without recurrence was not significantly different between the ESD group and the esophagectomy group. However, the esophagectomy group showed a higher overall adverse event rate than the ESD group (55.5% vs. 18.5%, *p* < 0.0001). In addition, more early adverse events occurred in the esophagectomy group than in the ESD group (48.2% vs. 8.9%, *p* < 0.0001). Accordingly, ER is a safe and less invasive treatment for subjects with pT1a esophageal SCC.

For pT1b cancers, ER is not sufficient given the considerable risk of metastasis, and additional treatments such as esophagectomy or chemoradiation are usually required. A multicenter study showed that the efficacy of ER followed by chemoradiation was comparable to that of primary esophagectomy and concluded that this treatment may be a standard treatment option for cT1b esophageal SCC [5]. In review articles [23,24], they concluded that ER-CRT may represent a valid alternative to radical esophagectomy, but caution must be taken for esophageal SCC with high-risk factors of metastasis such as deep submucosal invasion and lymphovascular involvement, as distant recurrence is not uncommon. Based on these reports, ER followed by chemoradiation may be a valid alternative treatment for patients without such high-risk factors. Further investigation is needed regarding the efficacy of this treatment for patients with high-risk factors of metastasis.

## 7. Future Perspectives

Increasing numbers of esophageal SCCs are now being treated with ER, in line with its expanded indications. However, it is necessary to overcome stricture after ER in order to further expand these indications. In particular, the high rate of stricture after resection of whole-circumferential lesions means that ER is sometimes avoided in patients with these lesions. Steroid therapy has been the mainstay for preventing stenosis, but it is necessary to further strengthen the steroid therapy or combine it with other methods to overcome stenosis after circumferential resection.

As the number of ER procedures increases, the number of patients who require additional treatment is also expected to increase, and the choice of additional treatment, e.g., esophagectomy or chemoradiotherapy, is a major clinical issue. Large-scale studies are therefore needed to compare these additional treatments and determine the optimal strategy.

## 8. Conclusions

Esophageal observation using NBI is recommended for the detection of superficial esophageal SCC. Preoperative workup by non-magnifying and magnifying endoscopy is a standard process for determining the invasion depth of superficial esophageal SCCs. Based on the Japanese ESD/EMR guidelines, clinical MM/SM1 carcinomas, except circumferential carcinoma, were an indication for ER. The curative effect of ER should be assessed based on histologic examination of the resected specimens. ER should be conducted based on a good understanding of the preoperative diagnosis, indication, curability assessment, and additional treatment of esophageal SCC.

## Figures and Tables

**Table 1:** Indications for endoscopic resection.

cT1a-EP/LPM non-circumferential lesion
cT1a EP/LPM N0M0 circumferential lesion ≤ 50 mm
cT1a MM/T1b SM1 cancer non-circumferential lesion

**Table 2:** Curability assessment.

Curative: pT1a-epithelial/lamina propria without lymphovascular invasion
Non-curative 1: pT1a MM without lymphovascular invasion
Non-curative 2: pT1b cancer invading the submucosa or lymphovascular invasion-positive

## Data Availability

Not applicable.
